# Interventional Treatment of Budd–Chiari Syndrome

**DOI:** 10.3390/diagnostics13081458

**Published:** 2023-04-18

**Authors:** Martin Rössle

**Affiliations:** Department of Gastroenterology, University Hospital, 79106 Freiburg, Germany; martin-roessle@t-online.de

**Keywords:** Budd–Chiari syndrome, TIPS, transjugular intrahepatic portosystemic shunt, angioplasty, interventional treatment, survival

## Abstract

Medical treatment is regarded as the primary course of action in patients with Budd–Chiari syndrome (BCS). Its efficacy, however, is limited, and most patients require interventional treatment during follow-up. Short-segment stenosis or the occlusion (the so-called web) of hepatic veins or the inferior vena cava are frequent in Asian countries. An angioplasty with or without stent implantation is the treatment of choice to restore hepatic and splanchnic blood flow. The long-segment thrombotic occlusion of hepatic veins, common in Western countries, is more severe and may require a portocaval shunting procedure to relieve hepatic and splanchnic congestion. Since it was first proposed in a publication in 1993, the transjugular intrahepatic portosystemic shunt (TIPS) has gained more and more attention, and in fact it has been so successful that previously utilized surgical shunts are only used for few patients for whom it does not work. Both interventional treatment options can be performed successfully in about 95% of patients even after the complete obliteration of the hepatic veins. The long-term patency of the TIPS, a considerable problem in its early years, has been improved with PTFE-covered stents. The complication rates of these interventions are low and the survival rate is excellent with five- and ten-year survival rates of 90% and 80%, respectively. Present treatment guidelines recommend a step-up approach indicating interventional treatment after the failure of medical treatment. However, this widely accepted algorithm has several points of contention, and early interventional treatment is proposed instead.

## 1. Introduction

Budd–Chiari syndrome (BCS) occurs in many forms, ranging from asymptomatic or mild disease to severe liver failure. The variability in the presentation of the disease depends on the extent of thrombosis, the velocity of its formation, and the presence and capacity of collaterals [1,2,3]. In addition to the hepatic veins, the portal vein as well as the inferior vena cava (IVC) may develop thromboses, which aggravate the disease and influence outcomes. With increasing collateralization, the congestion of the liver and intestine decreases, and the disease may change to follow a chronical course. The congestive state may be abbreviated and the damage reduced by interventional treatment, such as angioplasty in case of a short-segment (web-like) BCS, or portosystemic side-to-side shunts, which allow the transition of the portal vein into an outflow, thereby facilitating hepatic arterial perfusion.

Based on a study published in 2006 [4] a step-up therapeutic algorithm was proposed starting with medication, followed by angioplasty in patients with web-like BCS amenable to angioplasty including stenting, TIPS intervention, and, last of all, liver transplantation. Treatment escalation depends on non-response, which is defined by clinical and biochemical factors [4,5]. Medical treatment is essential for preventing further thrombus growth and formation. It is, however, ineffective with respect to the recanalization of hepatic veins, and 80–90% of patients do not show a clear and durable clinical response [6]. Most patients require an angioplasty or TIPS insertion, a fact which questions the treatment algorithm commonly presented by experts and consensus guidelines [4,6,7,8,9].

This report summarizes the technique and outcomes of the interventional treatment of BCS. The selection of patients and the timing of the interventions will be discussed in the light of the actual recommendations. A treatment algorithm is presented which counters the common guidelines and favours an early angioplasty or TIPS.

## 2. Interventions

### 2.1. Angioplasty

#### 2.1.1. Rationale

The recanalization of a short-segment occluded hepatic vein or the IVC may result in the restoration of the splanchnic and hepatic blood flow and lead to a clinical improvement. In the absence of cirrhosis, the procedure may be potentially curative [10].

#### 2.1.2. Technique

A percutaneous transluminal balloon angioplasty with or without stent implantation can be performed by a transjugular, transfemoral, or percutaneous transhepatic access. The technical success rates are over 90% for both hepatic veins and IVC webs [11,12,13,14,15,16,17,18,19]. After an angioplasty is performed and the stenosis or occlusion is resolved, the hepatic venous pressure gradient (wedged minus free hepatic vein pressure) should be determined and a transjugular biopsy should be performed at the same time. Both of these measures are important to better predict progression and exclude the earlier extension of the BCS into small hepatic veins and the presence of advanced fibrosis.

In patients with IVC webs, an angioplasty resulted in one-year primary and long-term secondary patency rates of >90% [12,14,18]. In hepatic vein web BCS, primary one-year and secondary long-term patency rates were 78% and 84%, respectively [16]. Complications were rare, appeared predominantly after a transhepatic access, and consisted of intraperitoneal bleeding (2%) and pulmonary embolisms (1%) [16].

The question of whether primary stent placement reduces the rate of restenosis has been addressed in a randomized controlled trial [20]. Forty-five patients received an angioplasty only and forty-three patients received an angioplasty plus a routine stent implantation. After the median follow-up of 27 months, the restenosis rate was 2% in the patients receiving a stent and 40% in the patients receiving an angioplasty alone (*p* < 0.0001). The three-year restenosis-free survival was 96% with stenting versus 60.4% without stenting. Authors concluded that routine stenting as a primary intervention may be advisable. It is effective and safe in the first-line treatment of short-length BCS.

### 2.2. TIPS

#### 2.2.1. Rationale

The rapid occlusion of the hepatic veins causes congestive liver damage due to reduced portal and arterial blood supply. In patients with a complete occlusion of all hepatic veins, hepatic blood flow depends on the development of intra- or extrahepatic collaterals which may discontinue disease progression and gradually change acute disease into chronical disease. If collateral formation is too slow or insufficient and does not increase blood flow adequately, the course of the disease may be progressive or deleterious, requiring immediate interventional or surgical treatment. In contrast to the liver, ischemia of the intestine is usually mild or absent due to the great distance between the occluded hepatic veins and the capillaries of the intestine [21].

As shown with surgical portosystemic shunts, hepatic blood flow and function can be readily improved by transforming the portal vein bed into an outflow tract for hepatic, splenic, and intestinal blood [22,23]. In the short term, both surgical shunts and TIPSs can immediately dissolve portal hypertension and intestinal congestion, improving ascites and preventing varices and variceal bleeding. In the long term, portosystemic shunts may also reduce or prevent the development of cirrhosis and regenerative hyperplasia [22,23]. Compared to surgical shunts, TIPS is less invasive and can be used in cases with the compression or occlusion of the IVC. As for many other indications, since their first application in patients with BCS in 1993 [24,25], the TIPS has almost completely replaced surgical shunt treatment.

**Figure 1:**
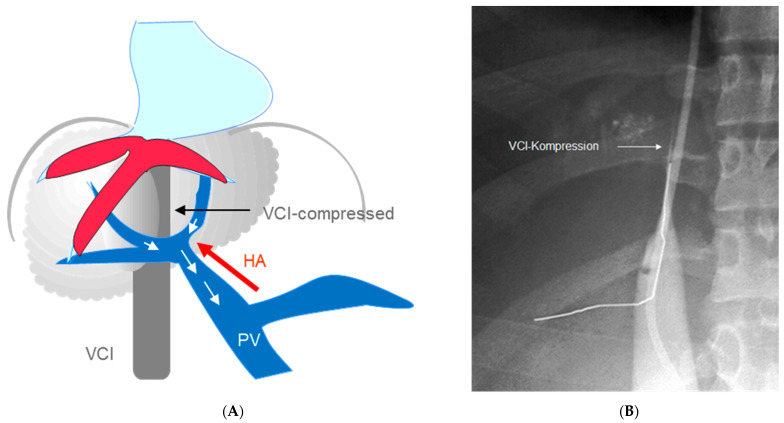
(**A**) Complete BCS. The portal vein is used as outflow tract. Enlarged caudate lobe leads to stenosis of the inferior caval vein; (**B**) Angiography of the inferior caval vein showing complete occlusion by the enlarged liver (VCI: inferior caval vein).

With respect to the enlargement of the caudate lobe, surgical porto-caval or meso-caval shunts alone were often ineffective and required an additional cavo-atrial anastomosis [22,23,26]. The TIPS, however, bypasses the stenosed IVC segment and operates with or without the enlargement of the caudate lobe (Figure 1).

#### 2.2.2. Technique of TIPS Implantation

**Figure 2:**
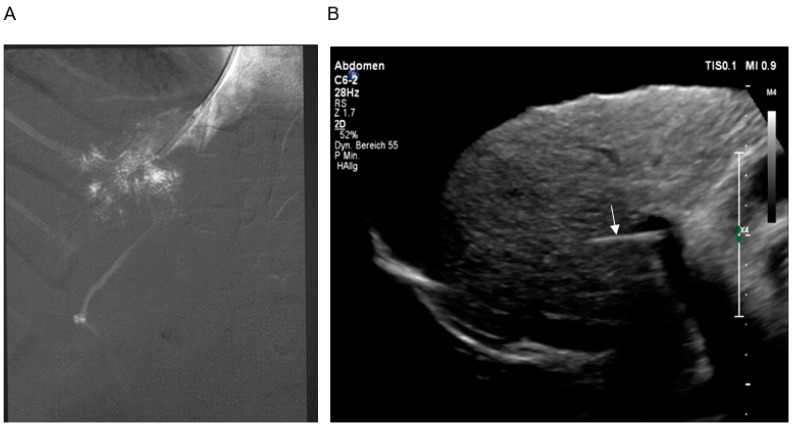
(**A**) Hand-injection of contrast into the parenchyma of the liver showing retrograde filling of a portal branch. (**B**) Sonographic guidance of the puncture. The location of the needle (arrow) and the portal vein is demonstrated.

**Figure 3:**
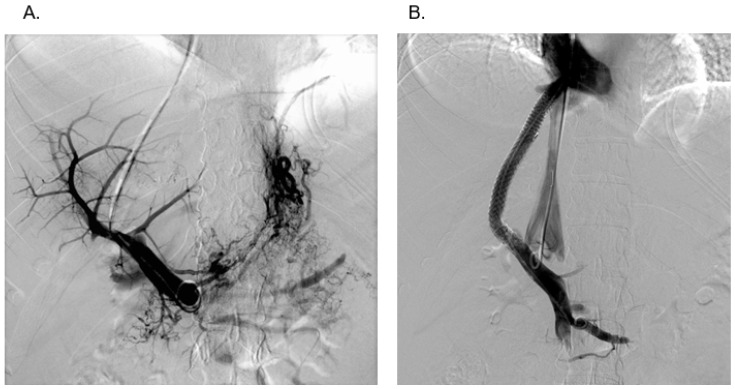
(**A**) Portography (DSA) after successful puncture of a very narrow right intrahepatic branch of the portal vein. (**B**) Simultaneous portography and cavography demonstrating good shunt function and perfect modelling of the two stents at both ends. This patient had only mild stenosis of the inferior caval vein by the enlarged caudate lobe.

The intervention begins with a transjugular cavography to show or exclude IVC involvement (Figure 1B). By moving the needle catheter assembly along the lateral wall of the IVC, a hepatic vein stump may be discovered and can be used to enter the hepatic parenchyma. About half of patients do not show remnants of hepatic veins, and, therefore, a puncture through the wall of the IVC is performed [27]. After having advanced the needle through the right lateral wall of the IVC, a small amount of diluted contrast confirms the intrahepatic position of the needle tip (Figure 2A). The next step is the puncture of the intrahepatic right portal branch which should be guided by transcostal sonography (Figure 2B). Sonographic guidance is important to reach a high level of technical success which may otherwise be limited by very small intrahepatic portal branches, which are often dislocated by the enlarged caudate lobe. A guidewire and catheter are now used to carry out angiographies and pressure measurements. The patency of the portal vein and the presence of varices may be demonstrated (Figure 3A). After the dilatation of the needle track, a covered stent is introduced and dilatated with a 10 mm balloon. It is strongly recommended to create a shunt with a diameter of at least 10 mm to achieve sufficient shunt flow and to relieve hepatic and intestinal congestion. Finally, angiography and pressure measurements are performed to control shunt function (Figure 3B).

At their time of diagnosis, a quarter of BCS patients have thromboses of the portal vein or IVC [28,29]. If the puncture process and the stent implantation are performed without complications, the portal vein catheter may be left in place and urokinase (100.000 U per hour, fibrinogen aimed at 120–150 mg/dL) and low-molecular-weight heparin may be administered. Daily angiographies are performed until patency is demonstrated, complications occur, or they continue to fail after 3–4 days of treatment [28,29].

The technical success of the TIPS intervention is 93% in experienced hands [28,29,30,31,32]. Portal vein thrombosis is not seen as a contraindication [29,31] and signs of clinical decompensation, such as tense ascites, jaundice, and hepatic encephalopathy, should be considered as reasons for urgent TIPS implantation [29,33,34]. Needless to say, only 10 mm PTFE-covered stents should be utilized to optimize flow and long-term patency [31,35,36,37].

In our previous study, technical complications occurred in 20% of patients and consisted of arterio-stent fistulae, intrahepatic hematoma, acute renal failure, and contrast dye-induced thyreotoxicosis [29]. With the exception of one patient with an arterio-stent fistula who required an occlusion of the feeding artery and an occlusion of the stent, no serious long-term complications persisted [29]. A multicenter study by Garcia-Pagan reported 17.7% had complications, and two resulted in death (one with an IVC injury, one with an infection). Most of the complications were bleeding (subcapsular, hemoperitoneum, biliary bleeding) [30]. Reversible procedure-related intraabdominal bleeding was also seen in three of fifty-one patients (6%) in a study by Qi [38]. Another complication is stent displacement or migration. The stent should be placed as demonstrated in Figure 3B. Displacement into the stem of the portal vein or close to the right atrium may complicate subsequent liver transplantation. However, using the Viatorr stent, dislocation or migration is very unlikely in experienced hands.

### 2.3. Pre- and Post-Interventional Management

Anticoagulation is mandatory as soon as a diagnosis of BCS is made. Heparin should be avoided since about 30% of patients have heparin antibodies at the onset of the disease [6,29]. This is why low-molecular-weight heparin is preferred. A phlebotomy and acetylic salicylic acid (100 mg/day) should be applied in patients with polyglobulia or thrombocytosis, respectively. Unspecific medication consisting of albumin, diuretics, dopamine, and antibiotics should be given as required.

**Figure 4:**
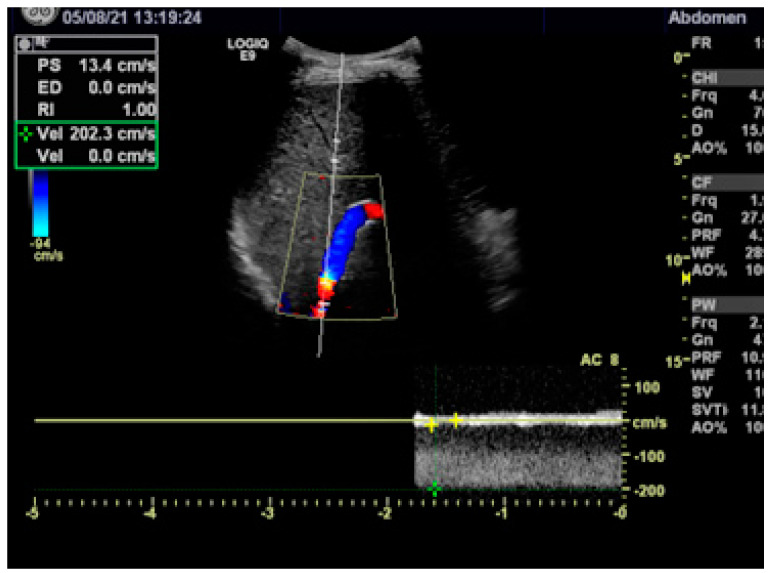
Duplex sonography showing stenosis in the proximal stent with a turbulent flow and a maximum flow velocity of 200 cm/s (2 m/s). According to the Bernouilli equation, the maximum flow velocity corresponds to a pressure gradient across the stenosis of 16 mmHg.

After a patient is discharged, anticoagulation has to be continued and a specific treatment of the hematological disease or coagulation disorder should be initiated. Duplex sonography before and every 3 to 6 months after a patient is discharged is recommended to assure the patency of the shunt and the portal vein. A reduced flow velocity of <60 cm/s or an increased flow velocity of >180 cm/s anywhere in the stent indicates shunt malfunction (Figure 4) [29,39]. In cases with a simple stenosis, the approximated Bernoulli equation (Δp = 4 v^2^) allows us to calculate the pressure gradient Δp in mmHg from the maximum flow velocity (in m/s) across the stenosis [39]. In the case presented in Figure 4, the maximum flow velocity of 2 m/s corresponds to an estimated pressure gradient of 16 mmHg, confirming the need for shunt revision. Shunt revision is also indicated when the flow velocity in the extrahepatic portal vein decreases to <30 cm/s. In addition to the duplex sonographic findings, any persistence of complications of portal hypertension (ascites, varices) should give rise to radiological shunt revision.

**Figure 5:**
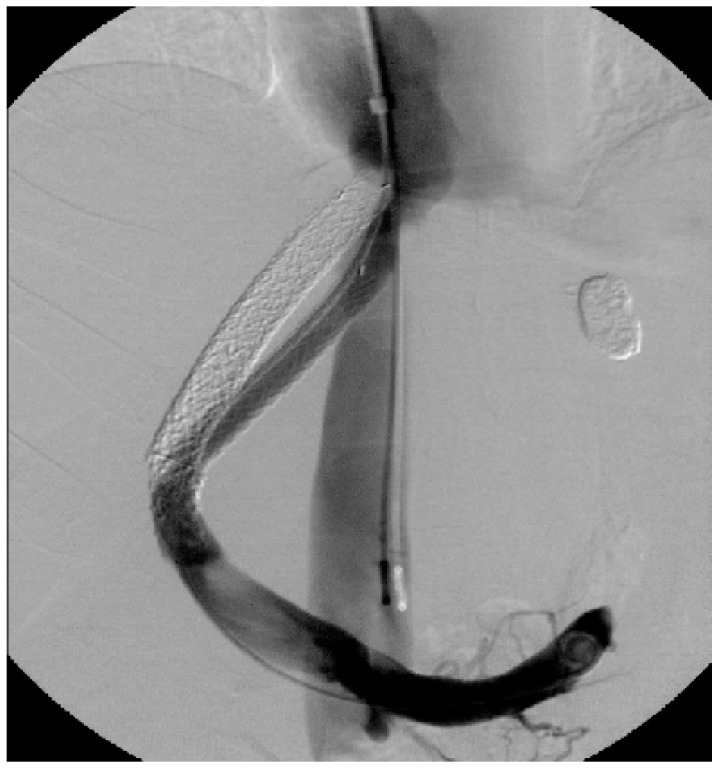
A 16-year-old female patient with fulminant Budd–Chiari syndrome due to essential thrombocytosis received a TIPS in 1998. Between 1998 and 2013, seven TIPS revisions were performed with implantation of additional stents with or without thrombolytic treatment. In 2013, the patient was admitted to the hospital for another revision after severe gastric variceal bleeding (see bucrylate clot). The catheterization of the occluded stent shunt was not possible and a transcaval puncture was performed for parallel stent implantation. The portography and simultaneous cavography (DSA) show good stent position and function. The shunt has been fully patent since then. Birth of a healthy girl in 2019.

Shunt failure is seen in about 25% of patients when covered stents are used [29,35,36,37]. The problem can be solved by stent-in-stent implantation or by parallel stenting, achieving secondary long-term patency rates of 95% [29,31]. A parallel second TIPS may be necessary if the stent cannot be entered, as demonstrated in Figure 5.

## 3. Results, Outcomes

### 3.1. Hemodynamics

**Figure 6:**
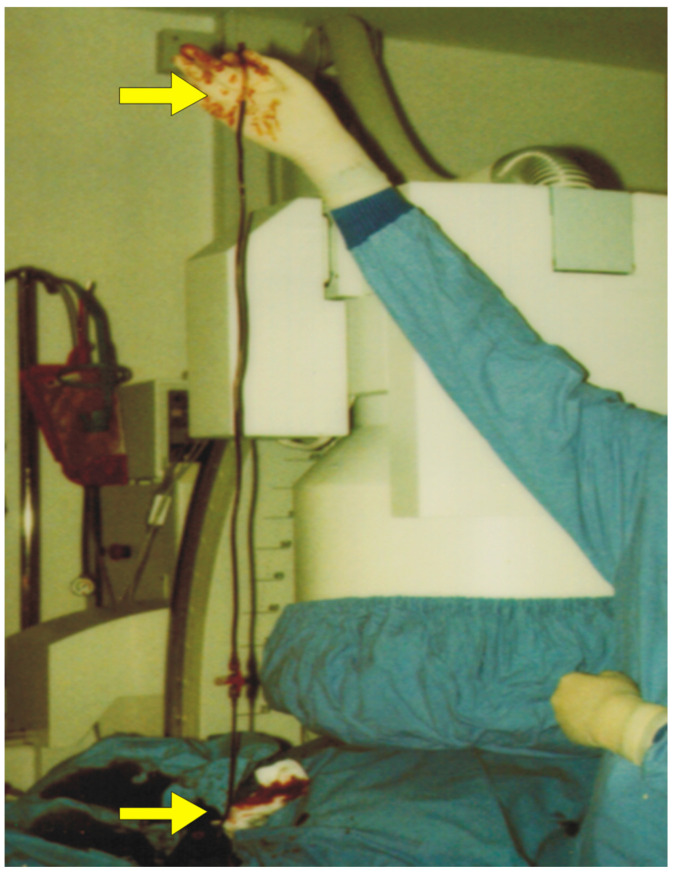
Example of a very high portal pressure in a patient with fulminant BCS. The height of the water column (between the yellow arrows) is 82 cm, corresponding to 63 mmHg. The value was confirmed by electronic measurement.

**Figure 7:**
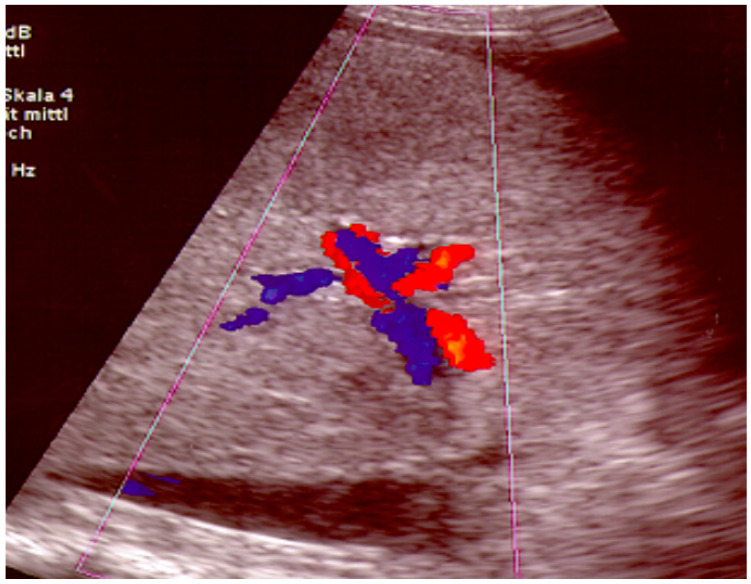
Intrahepatic portal flow (blue) in a patient with BCS after TIPS implantation. Hepatic artery in red.

In our single-center study including 59 patients with acute (4 weeks from diagnosis, 15 patients), subacute (6 months from diagnosis, 26 patients), and chronic BCS (18 patients), the portal pressures were highest in the acute group of patients (40.4 ± 10.1 mmHg) and somewhat lower in the subacute (33.3 ± 7.5 mmHg) and chronic disease (32.2 ± 8.9 mmHg) groups [31]. As demonstrated in Figure 6, acute/fulminant disease may result in extremely high portal pressures of up to almost 60 mmHg. The TIPS reduced the pressure gradient to 10.8 ± 4.9 mmHg. The shunt resulted in an increase in the blood flow velocity in the portal vein from 12.7 ± 10.0 cm/s to 48.6 ± 16.9 cm/s [29,31]. This was accompanied by an increase in the retrograde blood flow in the intrahepatic portal branches from +2 ± 11 cm/s to −11 ± 13 cm/s (Figure 7), giving clear evidence that the hepatic congestion was reduced or eliminated by the TIPS [29].

With respect to the systemic circulation, the TIPS improved the creatinine concentration within 2 weeks from 1.9 ± 1.7 to 0.8 ± 0.4 mg/dL [31]. This is surprising since, in contrast to cirrhosis, most patients with BCS do not express hypovolemia and only a few patients develop systemic vasodilation [40]. However, our study included 15 patients with acute BCS with liver failure and rapid development of severe ascites. These patients may have a greater risk of developing hepato-renal syndrome, which may improve after TIPS implantation [29,41].

### 3.2. Liver Function and Hepatic Encephalopathy (HE)

**Figure 8:**
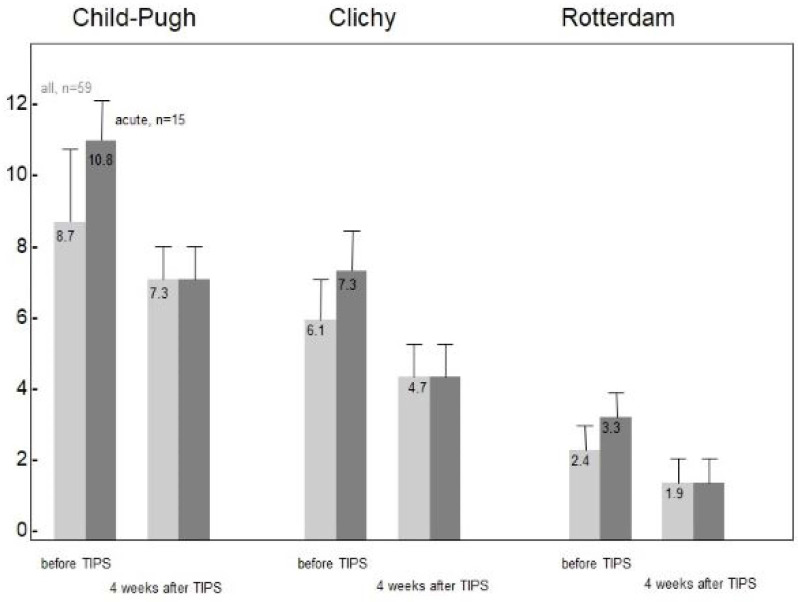
Development of Child–Pugh score, as well as Clichy and Rotterdam prognostic BCS indexes in 59 patients. TIPS improves the Child–Pugh score as well as the Clichy and the Rotterdam indexes significantly during the index hospital stay and thereafter. The effect was greatest in patients treated for acute or fulminant BCS (Adapted and modified with permission from Dr. T. Kappenschneider [31]).

With respect to biochemical variables, the TIPS improved hepatic and renal test results, almost reaching normal values within 2 weeks. This was, in particular, true for patients with acute or fulminant disease [29,31]. As shown in our study (Figure 8) and in other studies [30,36,41], the Child–Pugh score, the Clichy prognostic BCS index [42], and the Rotterdam prognostic BCS index [43] improved significantly after TIPS implantation. The effect was greatest in patients with acute disease.

HE was seen in 10% of patients before the TIPS intervention; all of them had fulminant disease [29]. Mild and reversible HE was seen in 0% [29] and 20% [30,38] of the patients after the TIPS implantation.

### 3.3. Survival

Survival may depend on the type of the BCS. In patients with short-segment BCS without a cirrhosis angioplasty with or without stenting may have a physiological restitution of the hepatic blood flow, resulting in excellent survival [11,12,13,14,15,16,17,18,19]. However, more than half of the patients required TIPS implantation during follow-up [38,41].

**Figure 9:**
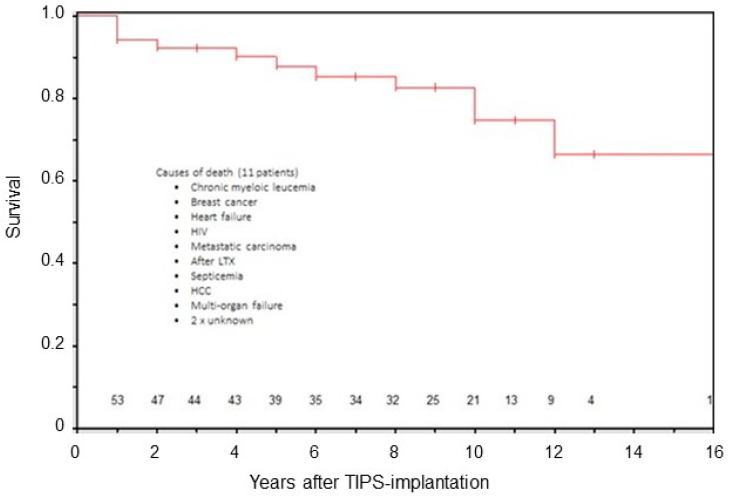
Survival of patients after TIPS implantation. Two patients were transplanted; one of them died.

Several studies have suggested that TIPS may improve survival [28,29,30,32,44,45,46,47,48], but comparable or randomized studies are lacking. A review on TIPS for BCS including 160 studies from 29 countries showed one-year and five-year survival rates of 80–100% and 74–78%, respectively [49]. The largest multicentre retrospective European study including 124 TIPS patients with long-term follow-ups [30] showed one-, five-, and ten-year OLT-free survival rates of 88%, 78%, and 70%, respectively. Survival cannot be predicted by the Rotterdam score [43] but it can be predicted by a new prognostic index (BCS PI TIPS), which includes age, bilirubin, and INR [30]. The same was true for the multicentre European study by Seijo et al. [41] which included 163 patients, 62 of whom had TIPS insertions. The one-, three-, and five-year survival rates in the TIPS patients were 88%, 83%, and 72%, and the BCS PI TIPS score [30] alone predicted survival. Our own study [31] including 59 patients showed comparable results with one-, five-, and ten-year transplant-free survival rates of 95, 90, and 80%, respectively. All of our patients had severe Budd–Chiari syndrome and were not responding to medical therapy. Most of the deaths were due to non-hepatic reasons (Figure 9). In accordance with the studies mentioned and with a study by Rautou et al. [46], Child–Pugh, Clichy, and Rotterdam scores were not valid to predict survival after TIPS, showing *p*-values of 0.84, 0.56, 0.75, respectively. In particular, 15 patients with acute or fulminant disease (transaminases > 10 times normal, severe ascites, hepatomegaly) showed a transplant-free 5-year survival of 91%, which is much different from the 42% prognosed by the Rotterdam score [31].

## 4. Discussion

An angioplasty may relieve hepatic congestion and normalize hepatic blood flow. However, its high rate of clinical failure (50%) that necessitates TIPS should indicate we need to better select patients. This can be achieved by measuring the hepatic venous pressure gradient (HVPG) beyond the web. In case of a normal HVPG, significant liver damage is unlikely, a TIPS is presumably unnecessary, and stent implantation to improve long-term patency after the angioplasty can be performed. In contrast, an elevated HVPG (>12–15 mmHg) indicates the presence of liver damage and questions the clinical efficacy of the angioplasty. Consequently, TIPS implantation may be the better choice, and implantation of a stent into the hepatic vein should not primarily be performed in order to avoid the technical difficulties of TIPS implantation. In addition to the measurement of the HVPG, a transjugular liver biopsy is recommended to quantify fibrosis and to support the decision for a TIPS.

Due to the very rare occurrence of BCS (about one per million), a comparison of treatments is difficult and randomized studies are not available. Considering the BCS Rotterdam score established for patients receiving medical treatment [43], TIPSs may lead to a 30% improvement in the five-year transplant-free survival rate in high-risk patients. In addition, patients not receiving TIPSs because of contraindications or technical failure revealed a low transplant-free overall survival rate of only 40% compared to 80% in similar patients receiving TIPSs [30]. In patients with acute BCS, TIPSs improved the apparent five-year survival (calculated by the Rotterdam score) by 50% (91% versus 42%) [29].

Surgical portosystemic shunts show an operative mortality of 5–32% and a five-year survival rate of 57 to 95%. Although there have been no comparative studies, the very low procedural mortality and the favorable five-year survival may argue in favor for the interventional treatment [7,23,29,50]. Both options have a relatively high stenosis rate of about 30% [45,51]. However, TIPS revisions are relatively safe and easy to perform and result in a very high secondary patency rate. The comparison of surgical shunts with medical treatment showed benefits in one study [52] but not in three other studies (42, 43, 50] when patients were allocated to comparable risk classes. A better result for patients with TIPS may, in particular, be expected in those with IVC obstruction [7,50]. Liver transplantation performed during the MELD era delivers results comparable to TIPS [29,53,54] with actuarial overall survival rates of 76–85%, 71%, and 68% at one year, five years, and ten years, respectively. However, patients were selected with very low “calculated” MELD scores of 7 to 20, influencing the result [29].

With respect to the timing of the interventions, guidelines and reviews recommend the step-up treatment according to non-response [6,7,8,9]. Based on a two-week evaluation, medical treatment was considered as a failure when ascites persisted, a negative sodium and water balance under a low-dose diuretic treatment could not be achieved, factor 5 remained below 50% of its normal value, and conjugated bilirubin did not decrease if initially high [5,7]. These criteria as well as the two-week evaluation interval may not meet the requirements of patients with acute or fulminant disease in which the treatment decision needs daily adjustments. In contrast to the strict step-up principle, an AASLD practical guideline suggested to check for a venous obstruction amenable for an angioplasty in all symptomatic patients right at the beginning and treat accordingly [55]. The step-up algorithm has also been criticized because it pays little attention to hemodynamics and to its possible improvement or even relief by interventional treatment. Thus, a new algorithm by Mancuso has suggested early TIPS treatment in patients with symptomatic (ascites, varices, liver failure) BCS [56,57].

In agreement with these suggestions, the stepwise treatment’s escalation may be criticized because of the following reasons:

First, the restoration of regular sinusoidal blood flow by the reopening of hepatic veins is extremely rare with medication [58] and does probably not justify a wait-and-see strategy.

Second, as demonstrated in a histopathological study, patients with BCS showed not only a high degree of venous outflow block but also obliterative changes in the intrahepatic portal vein branches [58]. Anticoagulation medicine given to all patients did not prevent venous obliteration. The changes in the portal veins, which are probably due to a reduced portal flow, may be prevented by a TIPS, which increases the backward blood flow in portal branches significantly [29].

Third, in patients with a web-like BCS, an angioplasty may restore regular hepatic blood flow, resulting in a cure. Therefore, appropriate patients should receive an angioplasty without delay. However, as demonstrated, more than half of patients required TIPSs during the follow-up [38,41].

Fourth, comparing spontaneous collaterals and TIPSs, the latter is superior with respect to timing and capacity. Both variants do not restore regular hepatic blood flow.

Fifth, as also shown in previous surgical studies, successful shunt treatment may stabilize liver histology and hepatocyte function and prevent disease progression to cirrhosis [22,23]. This has been substantiated by the rapid clinical and biochemical improvement in patients with acute or fulminant BCS after TIPS implantation [29].

Sixth, in candidates with a high risk of liver failure or variceal bleeding, early TIPSs may improve survival.

**Figure 10:**
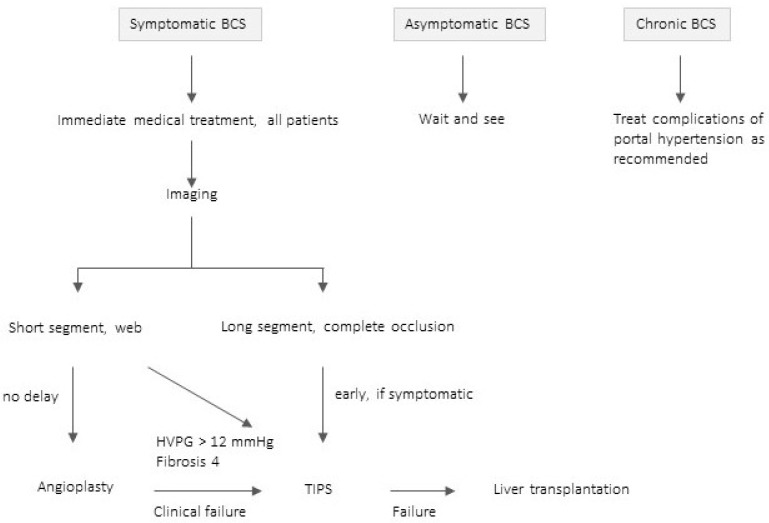
Proposal of a treatment algorithm and timing of interventions. Angioplasty should be performed without delay if a web-like BCS has been diagnosed. After successful angioplasty (demonstrated by lack of a significant gradient across the stenosis), the hepatic venous pressure gradient should be determined. An elevated gradient (>12 mmHg) should indicate the need for a transjugular liver biopsy, and, in case of advanced fibrosis, a TIPS should be implanted. The timing of the TIPS in patients with long-segment BCS may depend on the clinical severity of the disease. In symptomatic patients (liver failure, ascites, varices), early TIPSs may be indicated without waiting on their response to medical treatment.

The items presented strongly argue against the presently favored step-up algorithm. In accordance with Manusco [56,57], a new treatment algorithm is proposed which suggests early interventional treatment in symptomatic patients (Figure 10). This may further improve survival since liver failure and variceal bleeding, the major causes of death [41], can be treated or prevented effectively. The finding by Seijo et al. [41] that early or later TIPS implantation (before or after 1 to 3 months after diagnosis) did not influence survival has been regarded as a strong hint towards the stepwise strategy [8]. However, patients in this study were treated according to the stepwise algorithm in which those receiving the TIPSs earlier were worse than those treated later. This clearly suggests the presence of a selection bias, which causes doubt about this finding. Even more, the fact that survival was similar in patients treated early or late rather supports a positive effect of early TIPS implantation.

The question whether some patients with severe BCS may benefit from early liver transplantation without previous use of TIPSs remains unanswered. Up to now, there has been no reliable method to identify such patients [7]. Despite improving hepatic blood flow, the worsening of liver function after TIPSs may not be prevented in all patients. As shown previously [30], a few patients (7 out of 124 patients) worsened one year after the TIPS intervention, eventually requiring liver transplantation. The prognostic index, including age, bilirubin, and INR, identified these patients with a prognostic value of 88%, indicating that these patients should be listed for liver transplantation [30]. It should be pointed out that most prognostic scores include INR, which is a doubtful variable because it is influenced by treatment.

As also outlined in Figure 10, asymptomatic patients without biochemical and clinical abnormalities and without signs of portal hypertension may not require interventional treatment since congestion may be mild, absent, or compensated by other outflow routes. In patients with chronic BCS, the intention to prevent disease progression is secondary and the timing of interventional treatment may be dictated by the complications of portal hypertension. Having a thrombophilic disease, most patients with BCS need lifelong anticoagulation medicine, which is accompanied with an increased risk of variceal bleeding of up to 17–50% [7]. TIPSs may, therefore, be implanted earlier than usual, e.g., as a primary prophylaxis in patients with large varices.

In conclusion, the favorable results of interventional treatment of BCS may recommend its early use, which may further reduce early deaths due to liver failure. It may also be favorable to prevent and treat complications of portal hypertension, the leading cause of death during later follow-ups. The benefit of the recommended step-up strategy of waiting for the patient’s response to medical treatment is unproven and rather a matter of conjecture. In contrast, an angioplasty or TIPS delivers prompt and effective outflow, improving liver function, portal hypertension, and possibly one’s chance of survival.
